# Selections

**Published:** 1896-07

**Authors:** 


### Selections.

### REMEDY FOR LACERATION IN EXTRACTION.

IF any considerable amount of laceration has taken place during extraction, I attempt to replace the tissues by compressing with the fingers, or stitching into apposition any pendant portion of the gum, prescribing as a dressing,—

℞ Tannic acid, gr. xx;
Listerine, ℥iv;
Aquæ dest., ℥iv. M.
Sig.—Apply frequently to the wound.

—DR. OTTO ARNOLD, in *Ohio Dental Journal.*

